# The research activities of Ontario’s large community acute care hospitals: a scoping review

**DOI:** 10.1186/s12913-017-2517-4

**Published:** 2017-08-16

**Authors:** Giulio DiDiodato, John Alexander DiDiodato, Aidan Samuel McKee

**Affiliations:** 1 Department of Critical Care Medicine, Royal Victoria Regional Health Centre, 201 Georgian Drive, Barrie, ON L4M 6M2 Canada; 2 Eastview Secondary School, 421 Groves St. E, Barrie, ON L4M 5S1 Canada; 3 University of Western Ontario, 1151 Richmond St, London, ON N6A 3K7 Canada

**Keywords:** Knowledge translation, Acute care hospitals, Research activities

## Abstract

**Background:**

Ontario’s large community hospitals (LCHs) provide care to 65% of the province’s hospitalized patients, yet we know very little about their research activities. By searching for research publications from 2013 to 2015, we will describe the extent, type and collaborative nature of Ontario’s LCHs’ research activities.

**Methods:**

We conducted a scoping review by searching PubMed, Embase and the Cumulative Index to Nursing and Allied Health Literature databases from January 1, 2013 until December 31, 2015 for all publication types whose author(s) was affiliated with any of the 44 LCHs. Articles were screened and abstracted by three reviewers, independently. The data were charted and results described using summary statistics, scatter plots, and bar charts.

**Results:**

We included 798 publications from 39 LCHs and 454 authors. The median number of publications was 7 (Interquartile range (IQR) 23). Observational study design was most commonly reported in over 50% of publications. Program evaluation was the focus in 40% of publications. Primary LCH authorship was observed for 535 publications. Over 25% and 65% of the publications were attributable to 24 authors and 9 LCHs, respectively. There was minimal collaboration both within (21.2%) and between (7.8%) LCHs. LCH size and geographic proximity to academic hospitals had minimal impact on research activity.

**Conclusions:**

Ontario’s LCHs publish infrequently, collaborate infrequently, and their role in translational research activity is not well defined. A future survey questionnaire to LCH researchers identified through this review is planned to both validate and elicit their interpretations of our study findings and opinions about LCH involvement in research.

## Background

Ontario is Canada’s most populous province with over 13 million residents, and has a publicly funded and universally accessible hospital system that is administered by the provincial Ministry of Health and Long-Term Care (MOHLTC). Ontario’s acute care hospitals are classified as small community (<100 beds), large community (>100 beds) or academic hospitals by the MOHLTC [1]. There are 44 large community hospital corporations (LCHs) that range in size from 100 to 1232 average beds in operation (median 261, IQR 237) [2]. Compared to academic and small community hospitals, approximately 55% of all hospital beds are located in LCHs, and these LCHs are responsible for the care provided to over 65% of all medical and surgical patients annually [2]. Unlike academic hospitals, LCHs don’t have a mandate to conduct research as part of their operational activities. Consequently, a consortium of 18 acute care hospitals is conducting essentially all publicly funded acute healthcare research in Ontario [3]. This research model has unintentionally contributed to either failure or delays in the implementation of evidence into practice, and knowledge translation research initiatives have been initiated by both funding agencies and academic hospitals in their attempt to ameliorate this problem [4–6]. Most of these initiatives focus on funding groups that employ an integrated knowledge translation (iKT) research model [7].

The iKT research model has traditionally been described as involving ‘researchers’ who collaborate with ‘knowledge users’ to co-create evidence that will be more readily implemented into practice [7]. Apart from the potential to reduce the time lag between knowledge synthesis and practice implementation [8, 9] and reduce the discrepancy between treatment efficacy and effectiveness that is commonly observed in ‘real-world’ patients [10], there are many other good reasons why ‘knowledge users’ and their healthcare organizations should participate in research [11]. First, there is emerging evidence that patients whose healthcare providers or institutions participate in research experience better processes of care and improved outcomes [12–15]. Second, there is an evolving consensus that an increase in the implementation of evidence into practice will require the promotion of more practice-based evidence [16, 17].

To the best of our knowledge, the research activities of Ontario’s LCHs have never been described, and so we cannot fully describe the impact of iKT on community-based research [5]. In this study, we undertake a scoping review of the published research productivity of Ontario’s LCHs from 2013 to 2015. A scoping review has been defined as “a form of knowledge synthesis that addresses an exploratory research question… by systematically searching, selecting, and synthesizing existing knowledge [18–20].” The reason for our scoping review is to describe both the extent and type of published research activity being initiated and led by LCHs’ researchers, and to determine the extent of collaboration with both academic and non-academic centres. In addition, we aim to document any differences in research productivity that may be secondary to LCH characteristics, such as size or location, funding opportunities, extent of collaborative research activities, and other potential explanatory variables.

## Methods

A scoping review using the methodology described by Arksey and O’Malley [18], and refined by both Levac et al. [19], and Colquhoun et al. [20] will be used to address the research question. In general, a scoping review is an accepted method of knowledge synthesis using a pragmatic but systematic search strategy to answer an exploratory research question.

### Research question

For research articles published between 2013 and 2015 whose author(s) is affiliated with any of Ontario’s LCHs, what are the extent, type and collaborative nature of Ontario’s LCHs’ research activities?

### Search strategy and study selection

The search for research publications was limited to 3 years to ensure sufficient time periods to establish a trend, and establish a pragmatic limit to the number that needed to be reviewed. The LCHs included in the study had at least 100 beds in operation, and were not part of the Council of Academic Hospitals of Ontario during the study period, the current group of 18 hospitals designated as research centres and whose group contains only 1 large community hospital [3]. Conference abstracts, letters to the editor and book chapters were excluded, but all other publications were included to ensure that a comprehensive picture of research activity emerged. Both conference abstracts and book chapters are recognized as not having the same rigor of peer review as the other included publications, and this criterion alone was used to exclude them from inclusion in this study. The quality of the research publications was not evaluated, as is the norm for scoping reviews. Authorship order was dichotomized as follows: first, second or last author positions were deemed to have made a ‘significant’ contribution to the research and are defined as primary studies, while all other author positions were deemed to be of ‘lesser’ significance to the research and defined as secondary studies [21–25]. Author’s professional designation was not relevant to inclusion. Local LCH collaboration was defined as having two or more authors whose affiliations were from the same LCH listed in any position in the authorship order, whereas external LCH collaboration was defined as having two or more authors whose affiliations were from different LCHs listed in any position in the authorship order. In some circumstances, a research publication could have both local and external LCH collaboration. Types of publications were categorized as follows: editorial, observational study, experimental study, qualitative review, systematic review, guideline, or position paper.

PubMed, Embase and the Cumulative Index to Nursing and Allied Health Literature (CINAHL) databases were searched for research publications. One study investigator (GD) conducted a comprehensive literature search by using the following strategy: Step one: search each indexed database by the LCH name using the ‘affiliation’ field and the ‘year’ field to limit publications to 2013–2015. Step two: narrow the results by combining results from step one with the LCH address in the ‘affiliation’ field. Step three: for every LCH author, a modified ‘snowballing’ approach using the author’s full name was used to ensure maximal retrieval of all relevant publications. Step four: every publication was saved in Refworks® (http://www.refworks.com). Step five: all duplicates were removed. Step six: all the investigators independently screened each publication. All conference abstracts, letters to the editor and book chapters were discarded. Step seven: The screened lists of each investigator were compared to create the final list of relevant articles. If there was any disagreement between the lists, the publication(s) in question was included to ensure maximal sensitivity of the search.

### Data charting

All three investigators contributed to the identification of variables for extraction from the publications. An Excel® (www.microsoft.com) spreadsheet was created to support the collection of data. The variables identified for extraction included the following: full author name, LCH affiliation, journal name, year of publication, authorship position, corresponding author (yes/no), total number of authors listed, same LCH collaborator (yes/no), external LCH collaborator (yes/no), funded study (yes/no), publication type, and research focus. One investigator (JAD) reviewed the full-text publications and extracted all the data variables. The other two investigators (GD and AM) independently reviewed the full-text publications and edited the extracted data from the first investigator. The most senior investigator (GD) adjudicated any disagreement between the investigators’ extracted data.

### Data analysis

Summary statistics were used to describe the number of primary and secondary studies. Scatter plots and bar charts were used to demonstrate both relationships between and distributions of variables. χ^2^, ANOVA and Mood’s test were used for categorical, continuous and non-parametric group comparisons, respectively. STATA/MP 14.1 for Mac was used for all statistical analyses. Research ethics approval was not required as there were no human participants.

## Results

### Search results

**Fig. 1:**
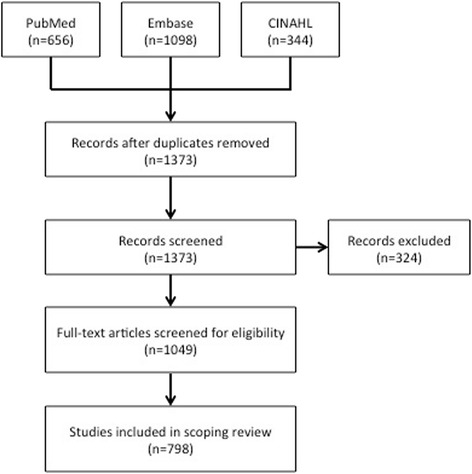
Flow Diagram of eligible studies

After duplicate publications were removed, the initial search strategy yielded 1373 publications. After the first screen, an additional 324 publications were removed, leaving 1049 publications for full text review (Fig. 1). The full text review resulted in the elimination of an additional 251 publications due to the following reasons; inability to retrieve the full text article (*N* = 49), and LCH was not in Ontario.

### Research publications

Of the 44 eligible LCHs, 39 LCHs published at least one paper over the study period. Thirty-seven LCHs produced 535 primary research publications, while 31 LCHs produced 263 secondary research publications over the study period. The mean and median number of total research publications for LCHs was 20.4 (standard deviation (sd) 29.4) and 7 (IQR 23), respectively, over the study period. The total number of publications increased over each calendar year (Table 1).

**Table 1:** Research publications by calendar year

Year	Publications		
	Total	Mean (sd)^a^	Median (IQR)^b^
2013	210	14.3 (9.4)	13 (19)
2014	279	23.6 (17.7)	18 (36)
2015	309	25.7 (17.4)	19 (39)

^a^One-way between year ANOVA F(2795) = 34.23, *p* < 0.001

^b^Mood’s Median χ^2^(2) = 17.89, *P* < 0.001

**Fig. 2:**
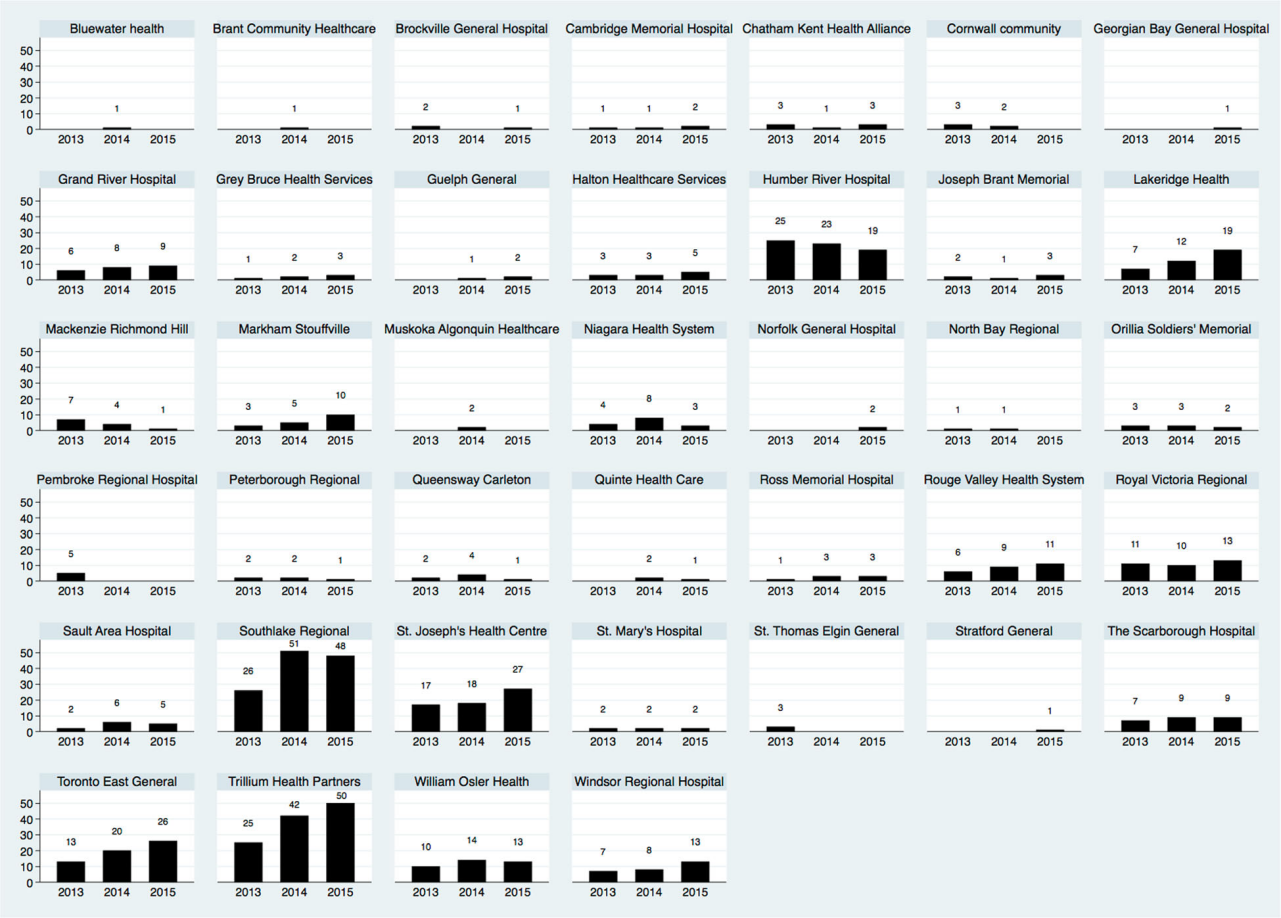
Distribution of publications by LCH

The distribution of publications across LCHs demonstrated significant heterogeneity (Fig. 2). The correlation coefficient between primary and secondary publications was 0.72, with each secondary publication resulting in an average increase in 1.3 publications per LCH (F(1,37) = 39.58, *p* < 0.001, 95% CI 0.9 to 1.7).

### Publication types and topics

The type and frequency of publication types did not change over the study period (Table 2).

**Table 2:** Publication types by year

Year	Publication Type^a,b^						
	1	2	3	4	5	6	7
	Publications (N, % Row Total)						
2013	16 (7.6)	9 (4.3)	5 (2.4)	139 (66.2)	2 (1)	25 (11.9)	14 (6.6)
2014	27 (9.7)	28 (10)	12 (4.3)	164 (58.8)	3 (1.1)	33 (11.8)	12 (4.3)
2015	26 (8.4)	32 (10.3)	11 (3.5)	184 (59.5)	1 (0.3)	43 (13.9)	12 (4.1)

^a^1 = Editorial; 2 = Experimental; 3 = Guideline; 4 = Observational; 5 = Position Paper; 6 = Qualitative Review; 7 = Systematic Review

^b^Pearson χ^2^(12) = 13.54, *p* = 0.331

Of the primary publications, 216 (40.4%) described local program evaluation and quality improvement activities. The most common publication topics were oncology (12%), cardiology (12%), nephrology (6.9%), infectious diseases (5.8%), rheumatology (4.6%) and psychiatry (4.4%).

### Authorship

There were 454 unique authors responsible for the 798 publications, with 330 unique authors responsible for the primary publications and 158 responsible for the secondary publications. There were 24 authors (5.3%) that had an uninterrupted continuous publication presence over the study period [26], accounting for 215 total publications (26.9%).

The mean and median number of authors per paper was 5.6 (sd 5.7) and 4 (IQR 5), respectively, with a range from 1 to 32 authors per paper. Authorship position ranged from 1 to 31, with 22 unique values. The distribution of first, second or last authorship position is shown in Table 3.

**Table 3:** Authorship position by year

Position	Year (N, % Row Total)^a^			Publications
	2013	2014	2015	Total
First	83 (29.6)	98 (35)	99 (35.4)	280
Second	37 (28.7)	43 (33.3)	49 (38)	129
Last	35 (27.8)	41 (32.5)	50 (39.7)	126

^a^Pearson χ^2^(4) = 0.77, *p* = 0.94

The correlation between authorship position and corresponding author identification was most significant for the first and last authorship positions (Table 4).

**Table 4:** Correlation between authorship position and corresponding author identification

Position	Corresponding Author (N, % of Row Total)^a^		
	No	Unknown	Yes
First	55 (19.6)	23 (8.2)	202 (72.2)
Second	106 (82.2)	11 (8.5)	12 (9.3)
Last	68 (54)	10 (7.9)	48 (38.1)

^a^Pearson χ^2^(4) = 160.7, *p* < 0.001

### Collaboration

**Fig. 3:**
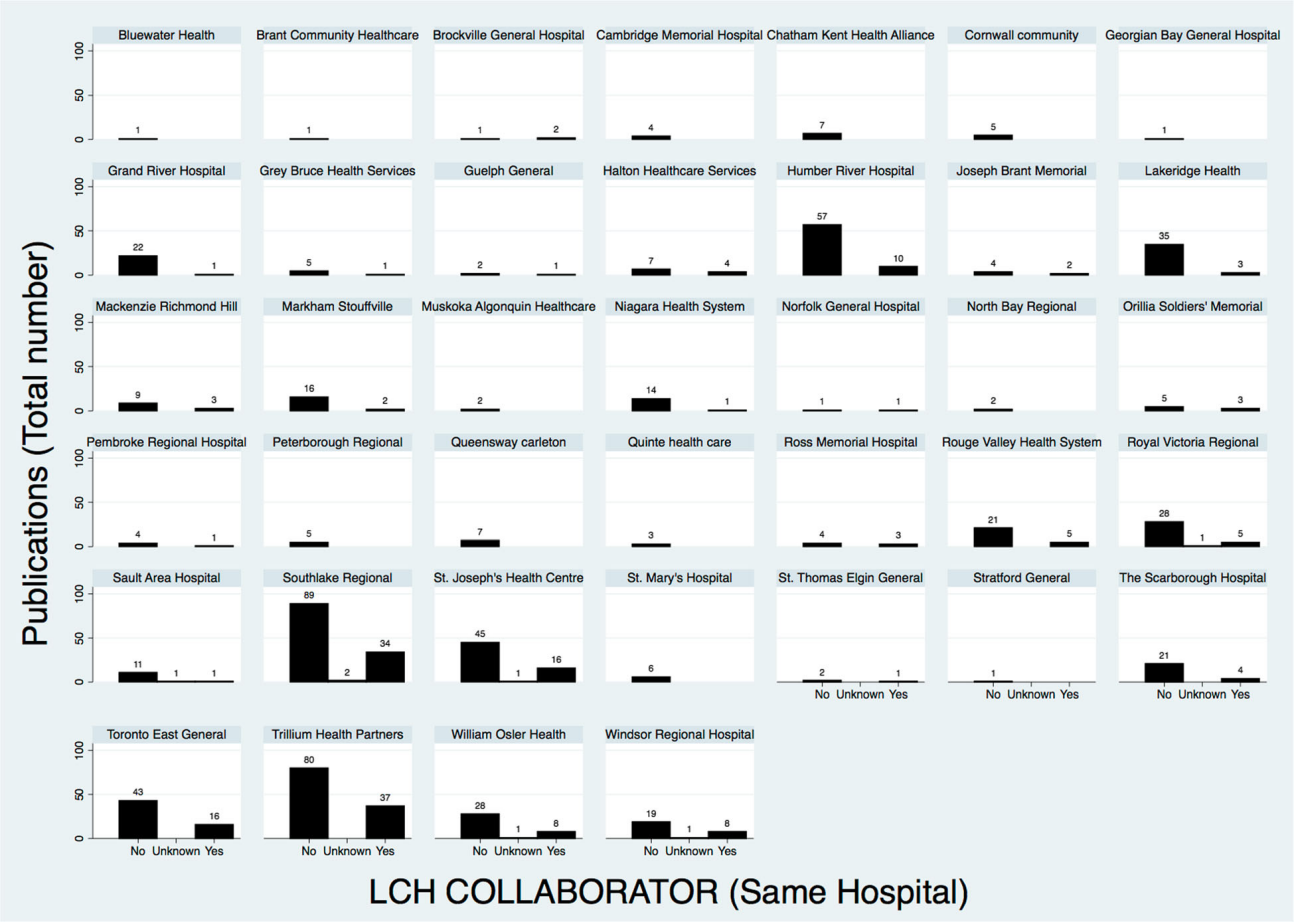
Distribution of collaborative research publications within the same LCH

**Fig. 4:**
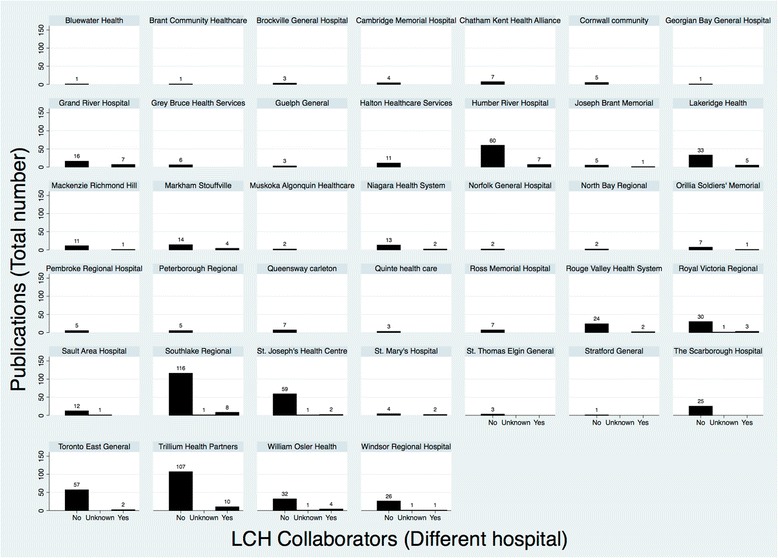
Distribution of collaborative research publications across different LCHs

Collaboration within the same LCH and between LCHs was relatively infrequent, occurring in 173 (21.7%) and 62 (7.8%) publications, respectively (Fig. 3 and Fig. 4). Fourteen (1.7%) publications demonstrated collaboration both within and between LCHs.

### Funding

Of the primary publications, 113 (21.2%) reported receiving funding from 79 unique funding sources. A search of the Canadian Research Information system database (http://webapps.cihr-irsc.gc.ca/cris/search, accessed June 1, 2016), using each unique author name (for the primary publications only), for all grants and funds (from Canada’s 12 largest research funding agencies) awarded to these researchers from 2010 to 2016 revealed that only 17 authors (3.7%) had received 24 grants (or 0.26% of the 9198 grants and funds awarded during this time period) totaling $36,965,857 (2010 CDN) (or 1.59% of the $2,325,721,614 total grants and funds awarded during this time period). These 17 authors were affiliated with 9 unique LCHs, and were listed as either co-investigators (16 grants) or principal investigators (8 grants). None of the LCHs were listed as the research site for these grants/funds. These 17 authors accounted for 170 (21.3%) of the total number of publications, and their 9 LCHs accounted for 537 (67.3%) publications.

### Hospital size and geographic location

**Fig. 5:**
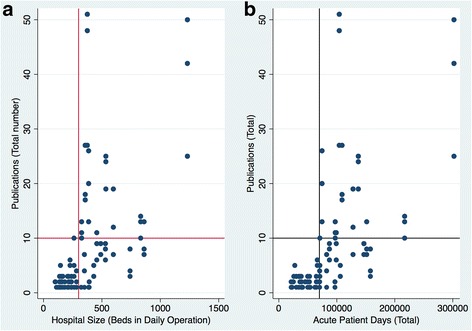
Relationship between research publications and **a** LCH bed size and **b** LCH acute patient days. The vertical line in (**a**) 300 beds and (**b**) 70,000 acute patient days

LCHs with fewer than 300 beds and 70,000 acute patient days seemed to consistently produce fewer than 10 publications over the study period, but there did not appear to be any consistent relationship between research productivity and either bed size or acute patient days in the larger LCHs (Fig. 5).

**Fig. 6:**
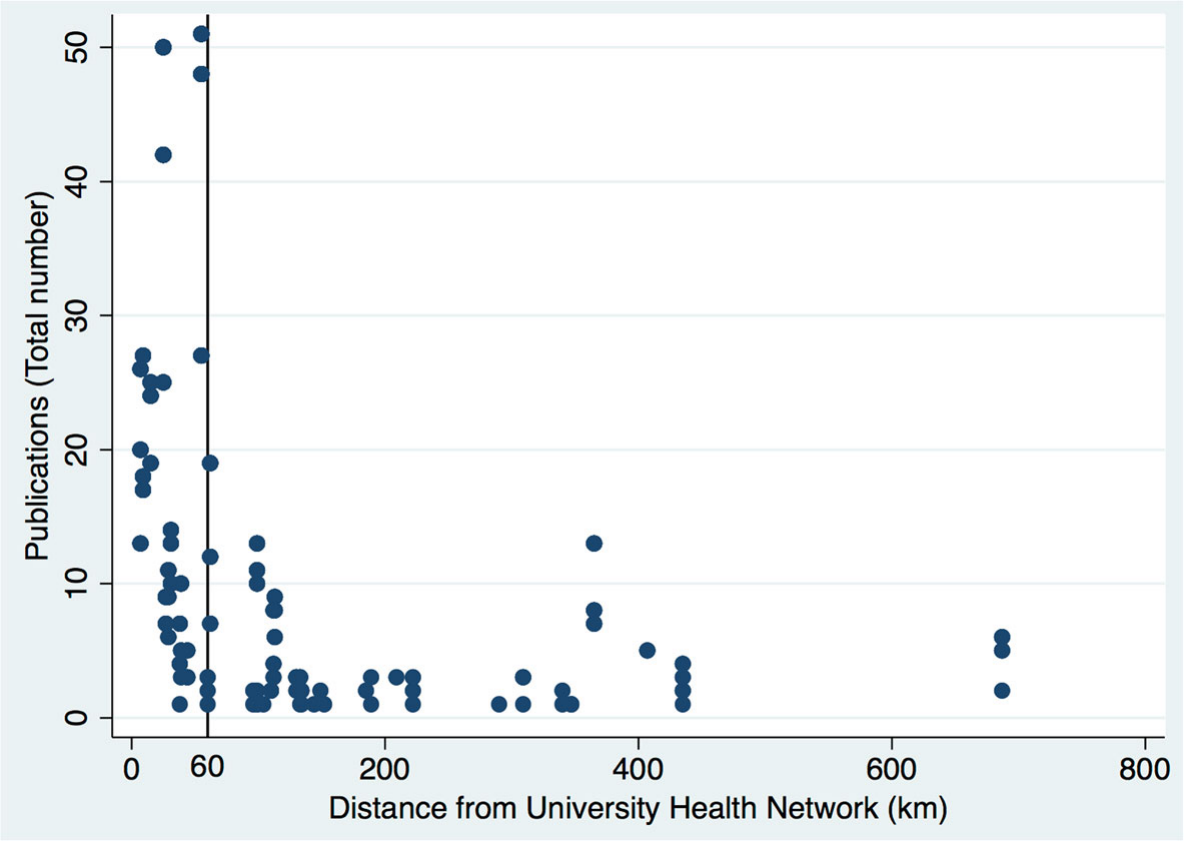
Research publications and geographic proximity to Toronto

Geographic proximity to academic centres was not correlated with research publications except for those LCHs close to Toronto, Canada’s largest city and healthcare network (Fig. 6).

## Discussion

Researchers affiliated with Ontario’s LCHs contributed to 798 research publications from 2013 to 2015, with 34% and 33% identified as the first author and/or corresponding author, respectively. While 40% of primary research publications were focused on locally relevant research questions, collaboration among researchers either within or between LCHs was infrequent, occurring less than 22% and 8% of the time, respectively. Most of the research publications were done by a core group of funded researchers affiliated with a few LCHs, a finding consistent with the general research community [26]. The scoping review doesn’t reveal whether these core researchers are embedded within their LCHs acting to build research capacity across the entire organization or whether they are simply ‘lone’ researchers collaborating with external partners to pursue their own academic research interests [27].

The influence of academic centres on research productivity was only evident for those LCHs located near Toronto, more likely reflecting the positive impact of that region’s population density, along with similar demographic profiles and healthcare infrastructures on facilitating the formation of research networks, an important criterion for promoting iKT [5, 28–32]. In addition, the largest LCHs were located near Toronto, suggesting that their increased participation in research was partly related to their size, a surrogate for increased research capacity.

The involvement of LCHs’ researchers as contributing authors in secondary publications was correlated with an increase in publications in which they were the principal investigator, suggesting that involvement in these research studies may have led to any increased capacity to undertake independent research activities by either the researcher or their LCH. However, this observation more likely represents an epiphenomenon whereby secondary publications are simply related to an increased tendency of certain LCHs or LCHs’ researchers to conduct research [29, 32].

This scoping review was mostly dependent on the accurate identification of authors’ affiliations to characterize the research activities of Ontario’s LCHs. Despite attempts to identify all relevant articles through multiple strategies, it is possible that we have underestimated the number of research publications involving Ontario’s LCHs [33]. However, given the consistency of findings from both year to year and within LCHs, and the comprehensiveness of the 3 indexed databases that were searched, it suggests our observations capture the vast majority of research publications associated with LCHs’ researchers, and more than adequately permitted us to describe the current research activities of Ontario’s LCHs and their researchers.

While the relationship between authorship order and investigator contribution remains open to interpretation [21–24], this study assumed a precedential and consistent approach to the assignment of primary and secondary research contributions, making conclusions from these classifications teleologically appropriate. We used the number of research publications as a surrogate for describing the research activity of Ontario’s LCHs and LCH researchers [33], recognizing that publication is an imperfect measure for research activity [34]. In addition, this scoping review excluded other sources of program evaluation and quality improvement [35], potentially underestimating the impact of iKT on healthcare services research. The study period was only 3 years, a time frame that should have adequately, albeit imperfectly, allowed us to describe a trend. While this study included multiple sites, the findings may not be relevant to other jurisdictions outside the province of Ontario.

In Canada, the Canadian Institute for Health Research (CIHR) is the largest public research funding agency. The CIHR have created three iKT funding opportunities: Partnerships for Health System Improvement, Knowledge Synthesis, and Knowledge to Action [29]. All three funding opportunities require that a researcher from a funding-eligible institution lead the project, but that they collaborate with community partners. Ideally, the collaboration should be structured to ensure that partners play an equal role in all aspects of the research study. Unfortunately, a recent qualitative evaluation of this approach suggested that these partnerships were rarely egalitarian; with 55% and 11% of community-based partners responding they had only an advisory capacity or a token role within the ‘collaborative’ partnership, respectively [29]. This is a consistent finding across other jurisdictions [30, 31]. While some Ontario studies have demonstrated the benefit of the iKT research model compared to end-of-grant knowledge translation models [36, 37], evaluations of the effectiveness of these iKT partnerships to improve knowledge translation has never been compared against any other model of community-initiated and community-led research projects conducted in the absence of academic partners, making any conclusions drawn by these funding agencies and academic hospitals highly susceptible to confirmation bias and invalid conclusions [5, 38].

Ontario’s LCHs are responsible for the majority of acute healthcare services provided to the province’s residents, yet these organizations seem to have minimal influential involvement in research activities; they publish rarely and have no access to public research funds or grants. What is not clear from this scoping review is whether these organizations want to be involved in research, and if they do, what do they aspire to achieve and how do they intend to do it? What is certain is that Ontario’s healthcare system needs these LCHs to become more engaged in effectiveness evaluations to ensure the sustainability of our Medicare system. How best to do this is unclear at this time. Most research agencies support academic-led iKT research models as a potential solution, but there are no clinical trials that demonstrate the superiority of this model compared to other models, such as embedding local researchers to support local research initiatives [27]. We suspect that each LCH may require a different approach, with LCHs with similar healthcare infrastructure and patient demographics to neighbouring academic centres utilizing a traditional iKT collaborative approach, whereas other LCHs in other regions without those academic relationships engaging local researchers and neighbouring LCH networks to achieve the same goals for their patients.

The optional last stage of a scoping review involves consulting stakeholders for their insight, and we intend to interview the 454 unique LCH authors identified in this study via a survey questionnaire regarding the validity of our data and their interpretations of our study findings and opinions about LCH involvement in research. By doing so we hope to understand how to promote, build and support current and future LCH research capacity.

## Conclusions

There are many drivers of poor medical care that compromise both patient outcomes and the sustainability of our healthcare systems. More research by the usual suspects supported by the same funding agencies is not the solution. What is needed is greater democratization of research funding and participation by patients and parts of the healthcare system that are currently excluded. Until that happens, no amount of tinkering with strategies such as iKT will succeed in reducing the research to practice gap.

## Abbreviations

CIHR: Canadian Institute for Health Research
CINAHL: Cumulative Index to Nursing and Allied Health Literature
iKT: Integrated knowledge translation
IQR: Interquartile range
LCHs: Large community hospitals
MOHLTC: Ministry of Health and Long Term Care
sd: Standard deviation
